# Diseases of the Stomach and Their Surgical Treatment

**Published:** 1902-03

**Authors:** 


**Diseases of the Stomach and their Surgical Treatment.** By A. W. Mayo Robson and B. G. A. Moynihan. Pp. xi., 308. London: Baillière, Tindall & Cox. 1901.

The Hunterian lectures of 1900 have been greatly expanded in the production of the present volume. Considerably more than one-third of the book is taken up by the consideration of gastric ulcer and its complications, and this is the best part of the work. As a consequence of this, other subjects have not been dealt with so fully as their importance deserves, though the book may be taken as an excellent summary of our knowledge of the surgery of the stomach.

The wide experience of the authors on the subject of gastric ulcer perhaps justifies the decided views enunciated on the treatment of acute hemorrhage by operation, views which are expressed firmly, clearly and rationally.

We are rather left to the conclusion that gastro-enterostomy should be performed in all cases of pyloric carcinoma in which pylorectomy is not justifiable; but when it is stated that of twenty-three such cases only thirteen recovered, and most of these for a comparatively short period (two only lived more than six months after operation), it would appear desirable that some discrimination should be adopted in the selection of cases for an operation which is only palliative at the best.

In several places the figures might, with advantage, be placed in closer proximity to the letterpress—thus, lymphadenoma is described on p. 55 and the illustration of it (fig. 12) appears on p. 57; and Senn's method of gastrostomy appears on p. 258, and the corresponding illustration (fig. 51) on p. 255.

The book is clear, readable and thoroughly sound in its teaching, and may be taken as a reliable exposition of the great advances which have been made in this subject.

